# An integrated statistical model for enhanced murine cardiomyocyte differentiation via optimized engagement of 3D extracellular matrices

**DOI:** 10.1038/srep18705

**Published:** 2015-12-21

**Authors:** Jangwook P. Jung, Dongjian Hu, Ibrahim J. Domian, Brenda M. Ogle

**Affiliations:** 1 Department of Biomedical Engineering, University of Minnesota – Twin Cities, Minneapolis, MN 55455, U.S.A; 2 Stem Cell Institute, University of Minnesota – Twin Cities, Minneapolis, MN 55455, U.S.A; 3 Cardiovascular Research Center, Massachusetts General Hospital & Harvard Medical School, Boston, MA 02114 U.S.A; 4 Masonic Cancer Center, University of Minnesota – Twin Cities, Minneapolis, MN 55455, U.S.A; 5 Lillehei Heart Institute, University of Minnesota – Twin Cities, Minneapolis, MN 55455, U.S.A; 6 Institute for Engineering in Medicine, University of Minnesota – Twin Cities, Minneapolis, MN 55455, U.S.A

## Abstract

The extracellular matrix (ECM) impacts stem cell differentiation, but identifying formulations supportive of differentiation is challenging in 3D models. Prior efforts involving combinatorial ECM arrays seemed intuitively advantageous. We propose an alternative that suggests reducing sample size and technological burden can be beneficial and accessible when coupled to design of experiments approaches. We predict optimized ECM formulations could augment differentiation of cardiomyocytes derived *in vitro*. We employed native chemical ligation to polymerize 3D poly (ethylene glycol) hydrogels under mild conditions while entrapping various combinations of ECM and murine induced pluripotent stem cells. Systematic optimization for cardiomyocyte differentiation yielded a predicted solution of 61%, 24%, and 15% of collagen type I, laminin-111, and fibronectin, respectively. This solution was confirmed by increased numbers of cardiac troponin T, α-myosin heavy chain and α-sarcomeric actinin-expressing cells relative to suboptimum solutions. Cardiomyocytes of composites exhibited connexin43 expression, appropriate contractile kinetics and intracellular calcium handling. Further, adding a modulator of adhesion, thrombospondin-1, abrogated cardiomyocyte differentiation. Thus, the integrated biomaterial platform statistically identified an ECM formulation best supportive of cardiomyocyte differentiation. In future, this formulation could be coupled with biochemical stimulation to improve functional maturation of cardiomyocytes derived *in vitro* or transplanted *in vivo*.

Differentiation of stem cells into specific lineages during development is tightly regulated by the local microenvironment including growth factors, extracellular matrix (ECM) proteins, and surrounding cells over time^1^,^2^. Stem cells taken outside the body are similarly dependent on the microenvironment and the past two decades have witnessed a surge in knowledge and corresponding technology to best manipulate stem cell state in a culture dish^2^. To date, the primary focus is manipulation of growth factor and small molecule delivery^3^,^4^. Progress in this area has been catalyzed by improved understanding of the genetic events that cause specification and the soluble factors that can stimulate these genetic events. In addition, it is technologically straightforward to add soluble components to a 2D culture system. More challenging is the identification and presentation of multiple insoluble components, namely ECM proteins, in 3D.

Differentiation of most cell types would benefit from the identification of defined, ECM formulations to enhance specification. Cardiomyocyte differentiation is an intriguing test case. Exogenous ECM proteins are required both to promote survival of cardiomyocytes *ex vivo*^5^ and to implement all cardiomyocyte differentiation protocols employed to date^2^. While the soluble components required to drive cardiomyocyte differentiation have become more well-defined (i.e., elimination of FBS and addition of MAPK inhibitor, PGI2, BMP2/4, Activin A, bFGF, Wnt3a, B27), the composition of exogenous ECM (i.e., matrigel or ECM of feeder cells) is largely unknown. In addition, stem cell-derived cardiomyocytes *in vitro* are currently unable to recapitulate all functional features of mature cardiomyocytes *in vivo*^1^. The feature most often lacking is contractility, a characteristic most definitely tied to the coupling of cell cytoskeleton with the ECM^6^,^7^,^8^. Hence, ECM certainly aids cardiomyocyte specification, but whether the maximum impact of ECM in this context has been realized in a culture dish is unclear.

Previous work developed and employed approaches to test the impact of multiple ECM formulations on (stem) cell behavior. First, an ECM microarray platform was developed wherein 2D combinatorial matrices could be spotted on a slide and seeded with cells. ECM were deposited with 32 different combinations of 5 different ECM molecules to identify combinations of ECM that synergistically impact hepatocyte function and embryonic stem cell differentiation^9^. Some years later, the same array concept was augmented to include pairwise combinations of 38 unique ECM for a total of 768 different combinations to show that fibronectin in combination with galectins-3 and -8 facilitates adhesion and metastatic progression of lung cancer cells^10^. Since then efforts have been made to employ 3D model systems to more closely approximate tissue-like scenarios. In particular, methacrylated gelatin^11^ and poly(ethylene glycol) (PEG)^12^ have been used as base material to incorporate discrete ECM combinations with cells in order to alter mesenchymal stem cell and embryonic stem cell behavior. The outcome in these cases is realized with more detailed analyses including principal component or network analysis.

While more combinations and higher throughput seems intuitively advantageous, we consider a few of the inherent limitations. First, there are technical challenges and specialized equipment associated with high throughput that renders the approach inaccessible to many laboratories. Second, even when the technology is available, multiplexing and small volume samples lend themselves to challenges of sample-to-sample variability. More importantly, statistical approaches employed to analyze microarray data to date are typically combinatorial covering discrete levels and only defined within the ranges tested in experiments. This analysis regime cannot lead to models of prediction based on varying inputs.

We propose an alternative that suggests reducing sample size and technological burden can be advantageous, especially when coupled to a Design of Experiments (DoE) statistical approach. Our laboratory has developed an approach that combines a physical culture platform with a statistical analysis regime to optimize differentiation of murine iPSCs to cardiomyocytes. In particular, we employ native chemical ligation (NCL)^13^ to polymerize 3D PEG hydrogels in aqueous and mild environments without utilizing initiators or UV-light. Since the NCL polymerization proceeds chemoselectively between the two precursors (PEG-Cys and PEG-thioester, Fig. 1a), a variety of ECM components and cells can be readily entrapped and the interaction of cells with ECM in this context is decidedly 3D in nature^14^,^15^. The chemoselective nature of the approach also makes precise control of various combinations of insoluble proteins in 3D possible while maintaining mechanical properties. Upon establishing this biomaterial platform, we employed DoE approaches to identify main effects and associated interactions that contribute to cardiomyocyte differentiation as determined by cardiac troponin T (cTnT) expression (Fig. 1b). Information acquired at this step was optimized with approximation of non-linear models, which identified a set of potential solutions from a formulation space not limited by the discrete set of experimental samples. Although DoE approaches have been widely performed in engineering and pharmaceutics^16^,^17^, combining modular biomaterial platforms and this statistical approach has been appreciated only recently^12^,^18^,^19^. With this approach, we found a formulation of multi-ECM matrices that included collagen type I, laminin-111, and fibronectin, significantly promoted the differentiation of murine iPSCs (miPSCs) into cTnT-expressing phenotypes without the addition of soluble factors in 21 days of culture (Fig. 1c).

## Results

### FEs to define the formulation for ECM-driven cardiomyocyte differentiation in 3D

**Figure 1:**
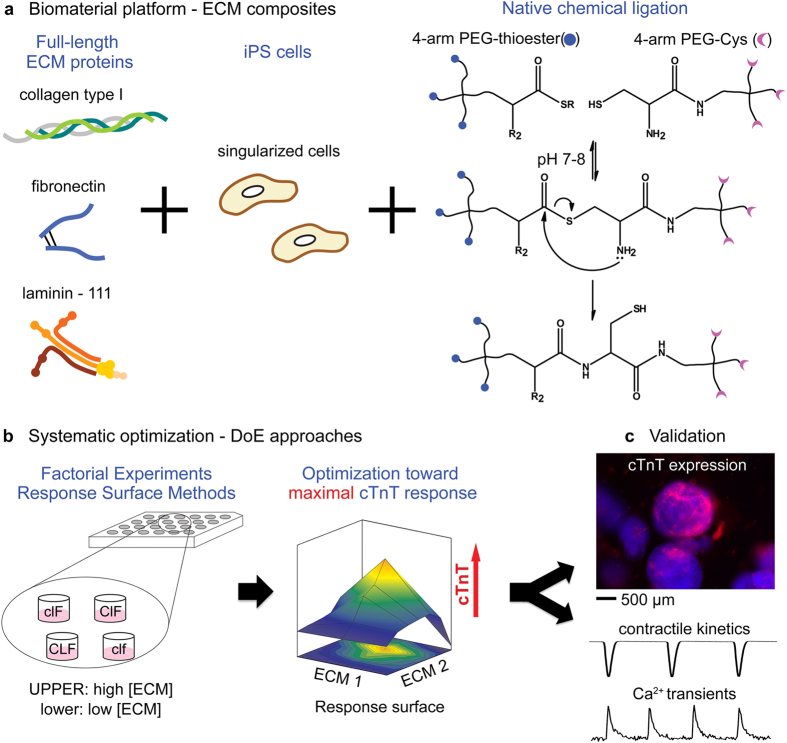
Integrating a biomaterial platform with statistical approaches. (**a**) Schematic representation showing production of ECM composites including several different ECM proteins at varied concentrations with single iPS cells via NCL of PEG precursors (thioester or Cys). We use three different ECM proteins representing fibrillar collagen type I, basement membrane laminin-111, and cell-ECM linking fibronectin, but a larger variety is technically feasible. (**b**) Statistical optimization of multiple ECM proteins can maximize the expression of cTnT by RSM, yielding a set of potentially optimal solutions. (**c**) The optimal formulation is validated by measuring the improvement of cTnT expression and the functionality of cardiomyocytes by imaging contractile kinetics and Ca^2+^ transients.

The first step of the DoE approach is termed factorial experiments (FEs), wherein each factor is set at two different levels for a given defined response. In our case, the FE factors were collagen type I (Col I), laminin-111 (LN) and fibronectin (FN) to be incorporated into PEG-ECM composites (Fig. 1) and the two levels were set to 0 mg/mL (low or −) and 0.83 mg/mL (high or + ), which ranged from 0 to 2.5 mg/mL of total ECM concentration in ECM composites (Table 1). These concentrations were selected for both practical and biologically motivated reasons. First, we found mechanical stiffness of the hydrogel remained unchanged with up to 2.5 mg/mL of protein at approximately 0.9 kPa (Supplementary Fig S1). The stiffness corresponds to the early embryonic heart^20^,^21^, which we reasoned could support differentiation of (mi)PSCs better than stiffer or adult-like mechanical environments. Thus in principle, any observed changes in differentiation behavior could be largely ascribed to the biochemical composition of ECM as opposed to the mechanical properties of the 3D scaffold. In addition, these three main effects (Col I, LN and FN) are used routinely but variably for cardiomyocyte differentiation protocols and are present in high volume fractions in the adult and developing heart^22^. Finally, we found that concentrations of ECM used in 2D culture^23^,^24^ (as extrapolated for the 3D volume space) would be included in the resultant formulation. Since we hypothesized that the responses would not be linear when each factor was changed from 0 mg/mL (low or −) to 0.83 mg/mL (high or + ) in FEs, a center point formulation (000 in Table 1) was added. The responses were defined initially as either expressed proteins indicative of cardiomyocyte differentiation (cTnT) or corresponding gene expression (Tnnt2).

**Figure 2:**
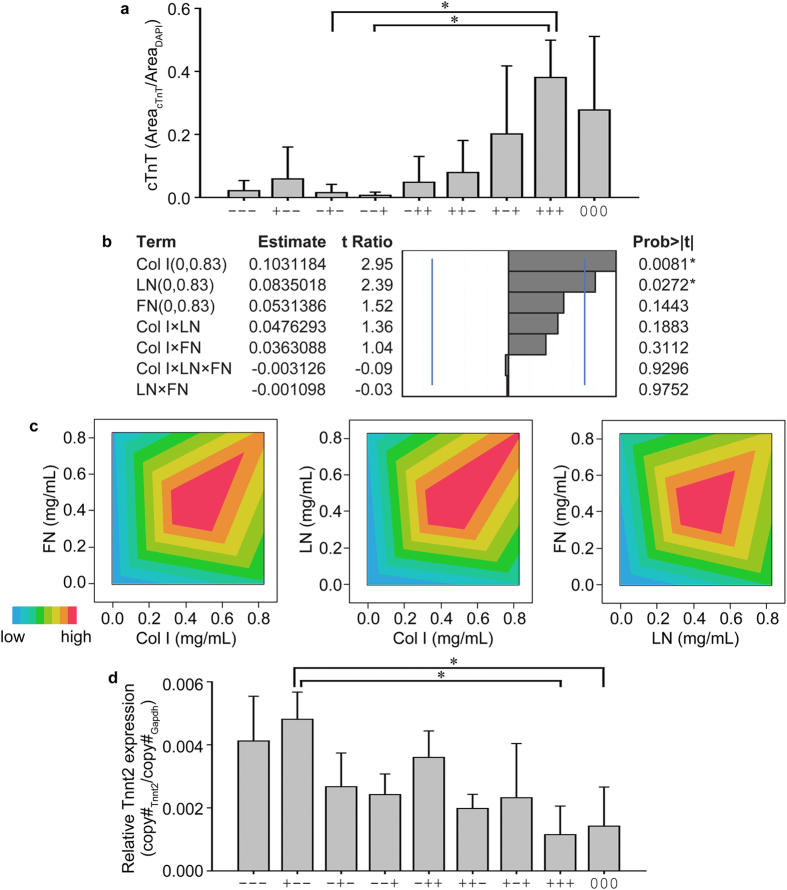
FEs inform the formulation space for three ECM proteins. (**a**) A simple plot shows that multiple ECM proteins (detailed formulations in Table 1) promoted the expression of cTnT, compared to no ECM (−−−) or single ECM (+−−, −+−, or −−+). (**b**) Effect magnitudes of all main effects and interactions for the expression of cTnT (protein). Blue lines indicate significant probability values of 0.05. (**c**) Contour plots showing the interactions between FN and Col I, LN and Col I, and FN and LN. (**d**) ECM composites as delineated in Table 1 were also tested for the expression of Tnnt2 (gene). Results in (**a**,**d**), ANOVA Tukey’s HSD *post hoc* test, n = 3, *p < 0.05, mean ± S.D.

FEs were analyzed up to the 3^rd^ order interactions with all three ECM proteins for both responses (cTnT and Tnnt2, response 1 and response 2, respectively). Conventionally, the responses can be plotted as a bar graph (Fig. 2a). It is logical to deduce that multiple ECM proteins increase the population of cTnT-expressing miPSCs and therefore cardiomyocyte differentiation. However, with this simple analysis, it is very challenging to distinguish the degree of impact on cTnT expression from individual ECM (i.e., the main effect) or the variation of main effects modified by other main effects (i.e., 2-way or 3-way interactions). From ANOVA Tukey’s *post hoc* tests, only three formulations were identified to be significantly different (Fig. 2a). However, by evaluating parameter estimates associated with DoE analyses, several main effects and interactions emerge (Fig. 2b). Col I and LN were significant main effects for maximizing cTnT expression and FN was positive to a less significant extent. The parameter estimates showed that once Col I was changed from 0 (low or −) to 0.83 mg/mL (high or + ), cTnT expression was improved by 0.103. This result means that the area fraction of cTnT-expressing miPSCs will be increased by 0.103 when Col I is increased from 0 to 0.83 mg/mL. In the same manner, addition of LN improves the area fraction of cTnT-expressing cells by 0.084. FN was a positive main effect to a lesser degree of significance in maximizing cTnT-expression from miPSCs. Since all interaction effects were below reasonable significance levels in the ranges in FEs, we graphed the interactions in contour plots (Fig. 2c) to glean further detail. Interactions between any two ECMs showed that concentrations around the center point 000 (0.42 mg/mL) were most synergistic. The higher interactions between Col I and LN were indicated beyond the upper boundaries (0.83 mg/mL) of Col I and LN. As a result, and as described below, the concentrations (levels) for Col I and LN were subsequently increased to 1.04 mg/mL, which was systematically augmented on top of the levels explored in FEs.

In addition to the expression of cTnT as an indicator of mature cardiomyocytes, RNA expression of cardiac troponin T (Tnnt2) was probed by real-time quantitative PCR (qRT-PCR) and was considered “response 2”. RNA was extracted from the entire ECM composite and copy number was normalized to Gapdh of each ECM composite (Fig. 2d). Parameters were all negative for improving Tnnt2 expression except the 2^nd^ order interaction from LN and FN (LN×FN) (Supplementary Fig S3). Interestingly, both LN and FN were *negative* and significant main effects for maximizing Tnnt2 expression. Given the striking difference in resultant parameters compared to cTnT protein expression, we probed the literature for evidence of Tnnt2 transcript dynamics with development and with accumulated cTnT protein in the cell. We found, expression of Tnnt2 (gene) peaks at postnatal days 8–10 with the subsequent decrease in expression until week 24 of fetal development^25^,^26^,^27^,^28^. These dynamics are quite interesting but render statistical interpretation quite complex relative to the continuously increasing (to maximum) nature of cTnT protein expression. For this reason, the cardiomyogenic response of miPSC to ECM composites was limited to cTnT expression (response 1) for all further experiments.

Thus, FEs were employed where main effects were set to range from the lowest to the highest possible concentrations of ECM proteins in a defined PEG hydrogel crosslinked with a chemoselective chemistry. Using a simple bar graph with ANOVA, the main effects and higher order interactions were not easily seen. In contrast, FEs identified not only the significant main effect and interactions of ECM proteins, but also the extent of those for maximizing our intended responses, the expression of cTnT.

### Response surface regression to hone the optimum ECM formulations for cardiomyocyte differentiation in 3D

In FEs, cTnT expression (response) was taken at two different levels (low or −, 0 mg/mL and high or + , 0.83 mg/mL) for each factor (ECM protein). Formulations containing more than two ECM proteins significantly enhanced the expression of cTnT, indicating each ECM contributed to enhance the expression of cTnT in synergistic or additive manners (Fig. 2). In order to reach a set of potential solutions to maximize the cTnT expression (response), we moved to the optimization with DoE approaches that allowed exploration of the region of interest by augmenting additional levels on top of FEs, called central composite design (CCD). The advantages of CCD is that no assumptions need to be made concerning the levels of factors (or structures of the factors), allowing one to analyze any set of continuous values for the factors^16^,^17^. In contrast to testing multiple discrete levels and generating array type data, this methodology leads to a set of predicted main effects from a non-linear continuous function, namely a “response surface” in equation (1). Typically, in industrial applications, quadratic components are sufficient to elucidate the curvilinear relationship between factors and responses, yielding a general model equation (*response surface* regression) with *k* factors:


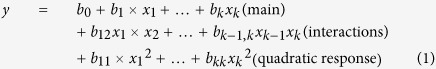


Following the design principle of creating CCD with the factors (Col I, LN, and FN) and the response (cTnT expression), additional levels (concentrations) were created as axial high (A, 1.04 mg/mL for Col I or LN) or axial low (a, 0 mg/mL for Col I or LN) corresponding to the highest and lowest levels for Col I or LN, respectively (Table 2). The levels were augmented on top of the previous FEs (Table 1) and scattered throughout the potential formulation space. Although the Col I and LN levels were expanded to go beyond what was tested in FEs, the role of FN needed to be refocused around the center point (000) of the FEs. Consequently, the highest level of FN was reduced to 0.42 mg/mL.

**Figure 3:**
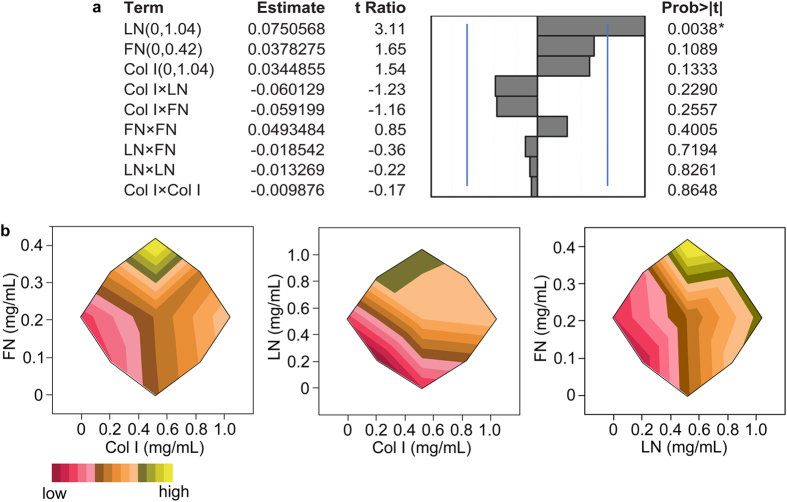
RSM of a formulation space modified to reflect adjusted levels (concentrations) of effects employed in the FE. (**a**) Effect magnitudes of all main effects and two-factor interactions to fit response surface regressions. Blue lines indicate significant probability values of 0.05. **(b)** Contour plots showing the interactions between FN and Col I, LN and Col I, and FN and LN from the RSM, which are different from those of the FE in Fig. 2c and indicative of different ranges tested in RSM.

From experimental runs by RSM (Table 2), all ECM proteins were positive for maximizing cTnT expression with varying extent (Fig. 3a). LN was the most significant main effect while Col I and FN were less significant. All of the 2^nd^ order interactions were identified negative, but the degree of extent was varying and insignificant to maximizing cTnT expression. Although this could lead to speculation of their antagonistic contributions, the 2^nd^ order interactions were not simplistic linear as depicted in Fig. 3b. FN showed the highest response around the maximum (0.42 mg/mL; axial high A of FN), while the interaction of FN with either Col I or LN identified positive as the concentration of Col I or LN increased (Fig. 3b). The extent of FN interactions with other ECM varies in wider ranges either being synergistic^29^ or antagonistic^10^, also depending on how FN is presented to cells (2D vs. 3D). LN reached a plateau level response above the mid-range in its interaction with Col I (Fig. 3b), which indicated that LN already reached an asymptotic value for cTnT expression. The increase of Col I enhanced the expression of cTnT in general throughout the range with either LN or FN. The second order interactions with the same main effect (Col I × Col I, LN × LN, and FN × FN) show the slope of the response surface. The matrix notation of the response surface is detailed in Supplementary Tables S1–3. The shape of eigenstructure was defined from the matrix of all second order estimates (Fig. 3a). Given in the first row of the canonical curvature table (Supplementary Table S2), eigenvalues of Col I, LN, and FN were 0.0617, 0.0138, and −0.0494, indicating the response surface of Col I and LN curves up from a local minimum and that of FN does the opposite from a local maximum. From these mixed eigenvalues, the response surface is saddle shaped, where the predicted solution of maximal cTnT expression was 1.15, 0.45, and 0.28 mg/mL of Col I, LN, and FN, respectively (Supplementary Table S3). We validated the predicted solution in three different ways by 1) assessing the expression of sarcomeric proteins of cells in ECM composites prepared using the predicted solution, 2) assessing contractile kinetics and intracellular Ca^2+^ transients of ECM composite-derived cardiomyocytes and 3) altering ECM content of composites with an adhesion modulator with impact on cardiomyogenesis.

### Model Validation 1: Confirmation of the results derived from RSM

**Figure 4:**
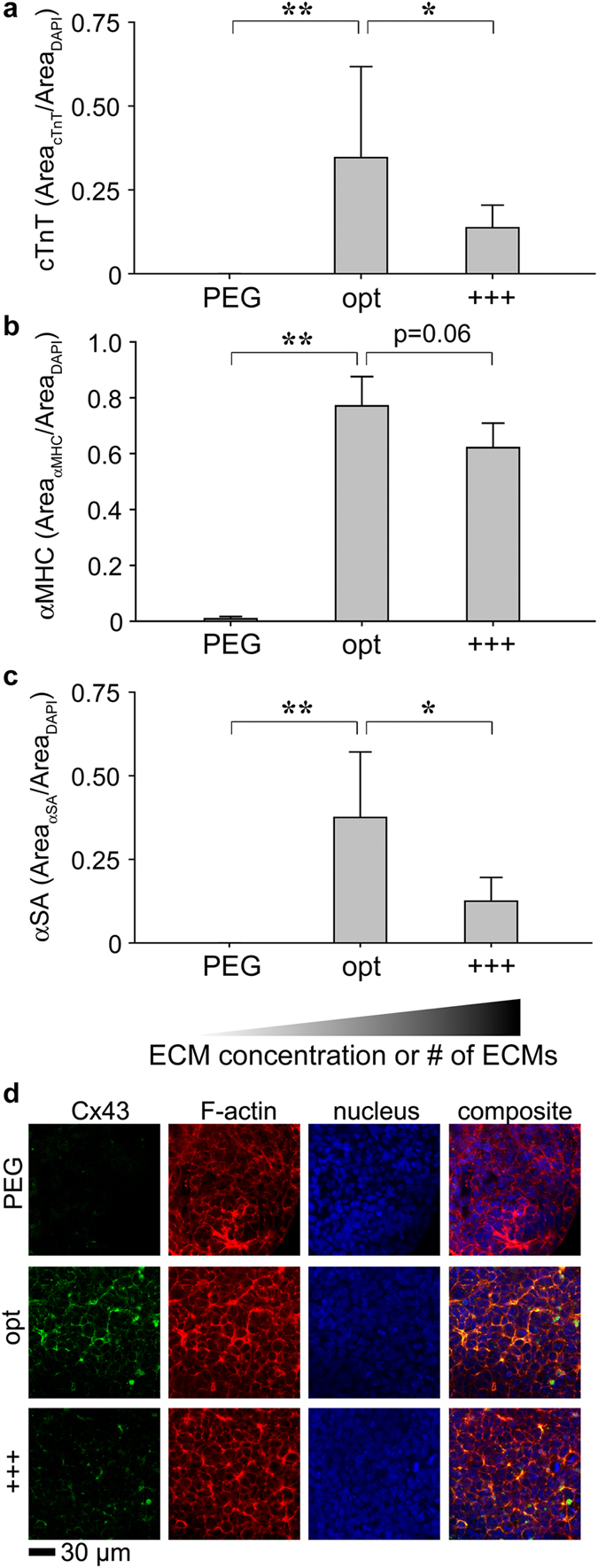
Validation of the optimized ECM formulation by the expression of sarcomeric proteins. Enhancement of cTnT (**a**) αMHC (**b**) and αSA (**c**) expression was tested with experimental (opt) and control groups (PEG and +++, formulations in Supplementary Table 4). (**d**) Expression of Cx43 was visualized with MPLSM in 3D ECM composites. ANOVA Fisher’s LSD *post hoc* test, n = 7 (**a**) or n = 3 (b or c) **p < 0.01 and *p < 0.05, mean ± S.D. The higher and darker gradient wedge indicates higher concentrations or numbers of ECM proteins associated in the formulation.

We tested whether the statistical optimization was indeed the “optimal” formulation to enhance the expression of cTnT. A suboptimal formulation (+++) from RSM and the no ECM control (PEG) were included (Supplementary Table 4). We found, the optimal formulation significantly enhanced the expression of cTnT over its primary contender, the +++ group (Fig. 4a) with the average percentage of cells expressing cTnT exceeding 35%. We also observed the enhancement of αMHC (α-myosin heavy chain, Fig. 4b) and αSA (α-sarcomeric actinin, Fig. 4c) protein expression from the optimal formulation. In addition to quantifying sarcomeric proteins, cells in ECM composites with the optimal formulation exhibited substantial Cx43 (connexin43)-positive staining (Fig. 4d), supporting direct electrical coupling of cardiomyocytes differentiated using this integrated biomaterial and statistical platform.

### Model Validation 2: Assessing contractile kinetics and imaging intracellular Ca^2+^ transients of cardiomyocytes

**Figure 5:**
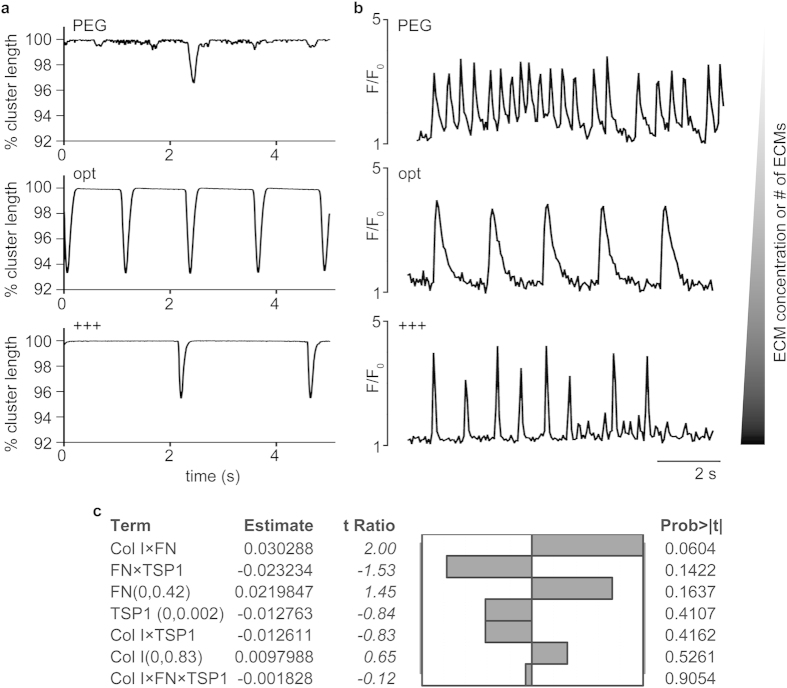
Validation of the optimized formulation by assessing contractility and Ca^2+^ transients and by adhesion modulation. (**a**) % cluster length was evaluated by utilizing high frame rate DIC. Representative contractility from ECM composites. (**b**) Fluo-4 AM (Ca^2+^ indicator) intensity (F) was normalized to the baseline fluorescence (F_0_). Representative local Ca^2+^ transient from ECM composites. (**c**) Effect magnitudes of all main effects and interactions for the expression of cTnT (protein) with Col I, FN, and TSP1 (formulations in Table 3).

To complement the phenotypic profiling of sarcomeric proteins, we utilized high frame rate (>150 frames per second) DIC imaging^30^ and confocal imaging of beating areas of ECM composites. The high frame rate methodology quantitatively assessed contractile kinetics via statistical analysis of movies of contracting cardiomyocytes wherein changes in cellular morphology over time are used to compute contractile kinetics. Spontaneously contracting cardiomyocytes cultured in PEG gels exhibited markedly reduced contractility with only rare cells achieving shortening of 3%. In contrast, cells cultured in optimized composites routinely exhibited shortening of 6–7%. In addition, shortening velocity was significantly higher in the optimized formulation relative to no ECM (PEG, *p < *0.05). To visualize excitation-contraction coupling of cardiomyocytes, we utilized fluorescent Ca^2+^ indicators and confocal imaging techniques, which provided the most versatile and widely used methods for analyzing cellular Ca^2+^ responses for different types of cells including differentiated PSCs to cardiomyocytes^31^,^32^. Cardiomyocytes differentiated for 7 days in EBs and matured for 3 days attached to a culture dish were used as a positive control (Supplementary Fig S4). Qualitative analysis of 30 s traces reveals apparent differences in Ca^2+^ handling between the groups (Fig. 5b; Supplementary videos 1–3). Quantitative analysis of interspike intervals (ISI) of optimal formulation (1.25 ± 0.35) showed a significant increase in ISI relative to the no ECM (PEG, 0.56 ± 0.19) control and an increase relative to the +++ formulation (0.86 ± 0.47) (Supplementary Fig S4a). PEG and +++ showed relatively fast and sporadic oscillation of fluorescence intensity (F) while the optimal formulation and the EB control showed rapid time to peak and slow, uniform decay. Of note, we do not draw comparison between the optimal formulation and the EB controls since the nature of signal propagation in 3D vs. 2D is quite different. This control was simply used as a means to garner a known positive signal. The relative maximum intensity of the fluorescent Ca^2+^ indicator (Fluo-4 AM) was also compared (Supplementary Fig. S4b). Maximum F/F_o_ ratios were relatively consistent between groups in spontaneously contracting composites.

### Model Validation 3: Evaluation of thrombospondin (TSP1), an adhesion modulator, on cardiomyocyte differentiation

To further validate the predicted solution, we incorporated TSP1 into our integrated statistical model. TSP1 has been shown to reduce cellular adhesion in the heart, especially in the context of pathology or scarring rather than regeneration^33^. In this validation step, the same FEs were generated with Col I, FN, and TSP1 while the levels (concentrations) of FN and TSP1 were modified (Table 3). Interestingly, none of the responses (cTnT expression) were significant and we did not observe any beating phenotypes throughout 21 days of culture. TSP1 did not contribute to improving cTnT-expression and the presence of TSP1 actually inhibited the response (cTnT-expression) (Fig. 5c). This change was not attributed to the diminished growth of miPSCs in TSP1-containing ECM composites as DAPI-stained areas were consistent between all DoE formulations (Supplementary Fig S5).

From these three different validation approaches, we found that the optimal formulation enhanced the expression of cTnT, αMHC, αSA and distinct Cx43 expression (validation 1) and the cardiomyocytes from the optimal formulation showed appropriate contractile kinetics and Ca^2+^ handling (validation 2). In addition, incorporation of a negative regulator of cardiac development abrogated cTnT-expressing and beating phenotypes (validation 3).

## Discussion

By integrating a novel 3D biomaterial platform and DoE approaches, we identified a formulation of multiple ECM proteins (Col I, LN and FN) capable of inducing significant levels of differentiation of cardiomyocytes from miPSCs without the addition of soluble factors. To perform DoE approaches, multiple ECM formulations were created by securing exogenous ECM in 3D space without modification or covalent linkage to the PEG scaffold. The chemoselective nature of the NCL crosslinking reaction allowed the use of multiple ECM proteins in wide ranges of concentrations without altering crosslinking chemistry for each ECM. Here, only simple mixing of ECM proteins in culture medium was necessary with appropriate pH adjustment to physiologic levels to account for the range of exogenous ECM proteins used and to ensure cell viability. Indeed, 3D ECM composites support the growth of encapsulated miPSCs over multiple weeks^15^. It is also important to note that instead of purifying populations of iPSCs or including sorted EBs in 3D gels^34^,^35^, singularized miPSCs were encapsulated in ECM composites and cultured without adding aforementioned soluble factors^2^,^3^,^36^. Although the 3D microenvironment itself (PEG hydrogel) was an important factor to support the expression of cTnT, formulations containing multiple ECM were significantly better than no ECM (−−−) or single ECM formulations (−+− or −−+or+−− in Fig. 2a).

To arrive at an optimum formulation (i.e., critical point) of multi-ECM, we employed DoE methodology to avoid testing the infinite number of combinatorial possibilities or performing conventional trial and error. Finding a critical point requires establishing a continuous function from independent variables, followed by differentiation of the continuous function to find the point at which the slope is zero. Here, those independent variables were ECM proteins (main effect) and the continuous function was generated to contain main effects, interactions of main effects and curvatures, which yielded a regression equation (1). With matrix operations of those variables in the response regression, the solution to maximize the expression of cTnT was found, resulting in 1.15, 0.45, and 0.28 mg/mL of Col I, LN and FN, respectively (approximately, 61%, 24%, and 15% of Col I, LN and FN, respectively). These concentrations or the relative ratio of ECM proteins, were obtained by an end-point analysis for promoting cTnT-expressing cells from miPSCs in a 3D ECM-incorporated scaffold over 21 days of culture.

Our results are difficult to compare with earlier studies wherein approximation^23^,^37^ and qualitative analyses^38^ were used to identify the relative content of ECM supportive of cardiomyogenesis. In addition, most *ex vivo* work has occurred in 2D *in vitro* systems^3^,^24^,^36^ and it is clear now that 2D and 3D culture regimes are quite distinct in terms of integrin engagement and associated cell fate signaling. For this reason, we have intentionally focused on progress in the field related to 3D *in vitro* models or *in vivo* studies. And while most studies are limited in their qualitative nature, a few quantitative studies are worth noting. First, in a 3D *in vitro* “interpenetrating network” model made of collagen, fibronectin and laminin, the authors find collagen matrices supplemented with small amounts of laminin (1.2 mg/mL collagen, 0.1 mg/mL laminin) were most supportive of cardiomyocyte beating^37^. Though only 10 formulations were studied and each was limited to two ECM components, the findings align broadly with ours. Second, an *in vivo* study of ventricular tissue during development, shows quantitative analysis of several ECM proteins including collagen types I and IV (basement membrane associated with laminin) and fibronectin^22^. The authors compare expression of each ECM component via immunofluorescence with development (i.e., from embryonic day 12.5 to postnatal day 2). It is interesting to note that at embryonic day 16.5 (the height of cell proliferation in the myocardium and differentiation to mature cardiomyocytes), the ratio of collagen (average arbitrary intensity units, a.i.u., 100), to fibronectin (40 a.i.u.) to basement membrane (50 a.i.u.) is quite similar to the critical value determined here. Thus our formulation might reflect late developmental conditions associated with cardiomyocyte differentiation and by exposing cardiac precursors to this formulation, we may provide the appropriate trigger to signal subsequent ECM production and remodeling needed for functional maturation^24^.

It remains to be seen whether continued improvements in cardiomyocyte differentiation and perhaps function *in vitro* could be realized via the inclusion of more obscure ECM types of the developing heart^39^. For example, Glypican-3 (Gpc3) is a heparin sulfate proteoglycan expressed widely during vertebrate development, where Gpc3-deficient mice display a delayed development of the coronary vascular plexus and an increased number of coronary arteries relative to the number of veins^40^. Versican is a chondroitin sulfate proteoglycan, the respective variants of which are expressed in specific spatiotemporal patterns during heart development^41^. Periostin is found in the developing heart and can interact with fibronectin, collagens type I and V, tenascin-C, and itself and acts as an adhesion molecule by binding integrins^42^. Finally, a novel protein called Abi3bp was recently shown to play an important role in regulating proliferation and differentiation of cardiac progenitor cells via integrin β1 and associated focal adhesion kinase phosphorylation^43^. The 3D model system and corresponding statistical approach described here are primed to enable investigation of the role of these smaller and less-well studied ECM proteins in cardiomyogenesis.

In this study, analysis of Ca^2+^ transients and contractility of cell populations was used to assess the extent of differentiation of cardiomyocytes in the optimum formulation compared to controls. The shape of the F/F_0_ traces was noticeably distinct between groups and the ISI was higher for cardiomyocytes developing in the optimized formulation relative to controls (Fig. 5b). In adult cardiomyocytes, a relatively small Ca^2+^ influx via voltage-activated L-type Ca^2+^ channels triggers greater amounts of sarcoplasmic reticulum (SR) Ca^2+^ release from the type-2 ryanodine receptor (RyR2) by the Ca^2+^ -induced Ca^2+^ release (CICR) mechanism. This leads to a rapid and high enough increase of intracellular Ca^2+^ concentration to initiate the interaction of contractile filaments and subsequent contraction (i.e., excitation-contraction coupling)^44^. Previous work showed that EB-derived cardiomyocytes from pluripotent cells possess heterogeneous calcium handling properties^45^ and that cardiomyocytes derived in this way were immature compared to murine ventricular cardiomyocytes with respect to the contractile properties related to the function of the SR and the inotropic response to β-adrenergic stimulation^45^. Of note, extended electrophysiological examination was not possible in this study without removing the cells from the composites, which we found to be detrimental to cell health. However, contractility studies were enabled via statistical analysis of movies^30^ of contracting cardiomyocyte of composites in 3D. Further approaches similar to these need to be developed to better determine whether functional maturation of cardiomyocytes is improved in 3D culture schemes. Further refinement of this integrated platform can also include multiple time points and the precise control of matrix-bound soluble factors to better appreciate the process of maturation and point of potential plateau.

In summary, the integrated biomaterial platform with DoE approach identified an optimal ECM formulation for differentiation of miPSCs to cardiomyocytes in the absence of stimulation by soluble factors. In the future, we envision this ECM formulation, and others derived in this way, will be used in conjunction with soluble factor stimulation in order to enhance the functional capacity of cardiomyocytes derived from PSCs. Potential improvements can be made by augmenting the variety of ECM proteins and matrix-bound factors included in composites, assessment of the kinetics of differentiation in ECM composites and inclusion of better means to assess functional maturity of derived cardiomyocytes. Broader advantages include the ability to apply this platform to the study of any cardiovascular cell type and associated matrix and to use identified formulations for basic studies and/or clinical tissue recovery.

## Methods

### Culture of murine induced pluripotent stem cells (miPSCs)

Murine induced pluripotent stem cells (miPSCs) were cultured using previously published protocols^15^. Embryoid bodies (EB) were generated using the hanging drop method *only* for the Ca^2+^ transient control. Briefly, resuspended miPSCs at 1.6×10^4^ cells/mL in culture medium without LIF and were deposited in 30 μL drops onto the lid of a 100 mm petri dish and suspended over 25 mL sterile PBS. Hanging drops were plated in culture medium without LIF onto gelatinized plates for 3 days until Ca^2+^ transient imaging.

### Formation of ECM composites and assembly of ECMs

Cysteine- and thioester-terminated 4-armed PEG macromonomers were synthesized using previously published protocols and singularized 3 × 10^5^ miPSCs/composite were encapsulated^15^. Either rat tail collagen type I (Col I, cat# 354236, Corning), mouse laminin (LN, cat# 354259, Corning), or human plasma fibronectin (FN, cat# 356008, Corning) or their mixture specified by the Design of Experiment (DoE) approaches was neutralized at 37 °C/5% CO_2_. Regardless of the formulation, the stiffness of composites was maintained at 0.9 kPa (storage modulus G′, Supplementary Fig S1). Since miPSCs were encapsulated and differentiated, 3D ECM scaffolds at the stiffness of early embryonic development^20^,^21^ would be more physiologically relevant than adult myocardium around or over 10 kPa^20^. ECM composites were kept in GMEM medium at 37 °C/5% CO_2_ for up to 21 days (medium change everyday).

### Extracting RNA from ECM composites

After 21 days of culture, ECM composites were collected in a microcentrifuge tube and stored at −80 °C with 10 μL of Trizol® (cat# 15596-026, Life Technologies) per ECM composite. When thawed, additional 30 μL of Trizol® was added to each ECM composite and mechanically disaggregated by micropestles. After ground ECM composites were allowed to stand for 5 min at room temperature, 8 μL of chloroform was added and vigorously shaken by hands for approximately 15 s. After 3 min at room temperature, samples were centrifuged at 12,000 g for 15 min at 4 °C, separating mixtures into three phases. Only a colorless upper aqueous phase was transferred into a new microcentrifuge tube. An equal volume of 70% ethanol (molecular biology grade) was added to each tube to make mixture of RNA in 35% ethanol. These mixtures were processed in PureLink RNA Mini Kit (cat# 12183-018A, Life Technologies) by following the suggested protocol.

### Quantitative real-time polymerase chain reactions (qRT-PCR)

Complementary DNA (cDNA) was synthesized following the instructions from the Maxima First Strand cDNA Synthesis Kit (cat# K1642, Thermo Scientific). Cardiac troponin T gene primer (Tnnt2) was purchased from Biorad (cat# 10025636). Primer efficiencies were extracted from RealPlex^2^ software and verified with melting curves. The gene expression level was determined as the copy number of Tnnt2 normalized to that of Gapdh.

### Imaging cTnT-, αMHC-, αSA- and Cx43-positive cells

After 21 days of culture, miPSCs in ECM composites were fixed with 4% paraformaldehyde for 15 min and extracted with 0.2% Triton-X 100 solution for 1 h at room temperature. Each step was performed on a rocker, followed by 5 min washing with PBS. Following a 2 h incubation at room temperature on a rocker with freshly prepared blocking solution (2.5 g of non-fat dry milk in 50 mL of 0.2% Trion-X 100 solution), rabbit anti-cTnT (cat# MS-295-P0, Thermo Scientific, 1:200 dilution in BGST buffer consisting of 1 g of Gly in 88 mL of water, 10 mL of 10× PBS, 5 g of BSA, 2 mL of goat serum, and 100 μL of Triton-X 100) antibodies were incubated at 4 °C overnight, which were probed by goat anti-rabbit Texas Red (cat# 31500, Pierce, 1:100 dilution in BGST) for 90 min. ECM composites were stored in 2.5% 1,4-diazabicyclo[2,2,2]octane (cat# D27802, Sigma-Aldrich) in 1:1 PBS and glycerol with 1 μg/mL 4′, 6-diamidino-2-phenylindole (cat# D9542, Sigma-Aldrich) until imaging. ECM composites were imaged with an Olympus IX81ZDC microscope equipped with 10× (NA = 0.25) objective lens using MetaMorph® slide scan function to acquire 8 by 9 fields of view per ECM composite (Supplementary Fig S2). The ratio of the cTnT-positive area to DAPI-stained area was measured with FIJI software. Sarcomeric proteins were probed by primary antibodies (all primary antibodies were diluted in 1:100 in 5% donkey serum, incubated at 4 °C overnight) against Cx43 (cat# sc-9059, Santa Cruz), αMHC (cat# sc-32732, Santa Cruz), and αSA (cat#A-7811, Sigma-Aldrich), which were probed by donkey anti-rabbit FITC, cat# 711-545-152, Jackson Immuno Research Lab, 1:200 dilution in 5% donkey serum, incubated for 1 h). After washing with PBS, F-actin was probed with TRITC-conjugated phalloidin (cat# FAK100, EMD Millipore, 1:100 dilution in 5% donkey serum, incubated for 45 min). Cx43-positive cells were visualized with a 60× (NA = 1.0) objective lens by using a mode-locked Ti:Sapphire laser on a multi-photon laser scanning microscope (MPLSM, Bruker Nano).

### Design of Experiments (DoE) approaches and statistical analyses

The efficient investigation of the relative ratio of multiple ECM proteins directing the differentiation of miPSCs into cTnT-positive phenotypes were facilitated by the chemoselective nature of the NCL of PEG hydrogels. The work proceeded in two stages: 1) Factorial Experiments (FEs) with multiple ECM protein to determine how significantly each ECM protein impacted the differentiation of miPSCs into cTnT-positive phenotypes and whether any response to a particular ECM was dependent on the level (concentration) of the other ECM proteins (i.e. interactions between main effects) and 2) determining an optimal relative ratio of multiple ECM protein formulation using response surface methodology (RSM).

In the first stage, a two-level, three-factor (2^3^) design was utilized, in which each factor (Col I, LN, and FN) was set at a high (0.83 mg/mL) or low-level (0 mg/mL), producing the matrix of experimental runs shown in Table 1. This design allowed for the identification of the most significant factors and the identification of interactions among those factors. JMP software (SAS, North Carolina, USA) was utilized to facilitate the generation of this experimental matrix, which consisted of eight (2^3^) different formulations and one center point (000) that can be added if the factors in the design and the responses are suspected to have curvilinear relationship. ECM composites were produced and analyzed in triplicate for two different responses, cTnT protein expression and Tnnt2 gene expression, amounting to 54 total gels. JMP software was utilized to fit the data, conduct ANOVA to determine significance, sort parameter estimates, and identify statistically significant factors and interactions.

In the second stage, RSM experiments were employed to determine a potential optimal formulation of multiple ECM proteins. The RSM experiments explored different levels and ranges of ECM proteins in the ECM composites, utilizing a central composite design (CCD, Table 2). This design is consisted of 15 different combinations of three different factors (Col I, LN, and FN), in five different levels. The CCD formulations included Col I and LN ranging from low axial point (a, 0 mg/mL) to high axial point (A, 1.04 mg/mL) and FN from low axial point (a, 0 mg/mL) to high axial point (A, 0.42 mg/mL). In CCD formulations, one center point (000) was included due to the quadratic nature of RSM. The response was fixed to cTnT expression throughout RSM experimental runs (a total of 45 gels). JMP software was utilized to fit the data, conduct ANOVA and lack-of-fit analysis for determining significance and reproducibility, sort parameter estimates, and identify the predicted solutions from the response surface.

In order to assess the impact of matricellular proteins on cTnT-expressing phenotypes, the FE was repeated in the similar method described above with different formulations and levels (Table 3). The level (concentration) of thrombospondin-1 (TSP1) was calculated from the surface concentration required for coatings^46^ and transformed to a volume density to produce an ECM composite.

For all other non-DOE approaches, one-way ANOVA with Fisher’s LSD or Tukey’s HSD *post hoc* tests for multiple comparisons was performed, where p values <0.05 were considered significant. At least three independent experiments were performed.

### High frame rate DIC and confocal Ca^2+^ transient imaging

After 21 days of culture, high frame rate DIC microscopy was performed with a Nikon TiE Deconvolution microscope equipped with 10× (NA = 0.25) objective and Ibidi live cell environmental chamber at least 150 frames per second (fps) for 30 s. Contractile kinetics of differentiated miPSCs was calculated using the Baseline Adjusted Similarity Comparison (BASiC) curve methodology^30^. ECM composites were loaded with 5 μM Fluo-4 AM (cat# F14217, Life Technologies) in serum-free GMEM. After incubating at 37 °C/5% CO_2_ for 30 min, ECM composites were incubated for 30 min with Tyrode’s salt solution at 37 °C/5% CO_2_ to allow de-esterification of intracellular AM esters. Intracellular calcium transients were recorded with a Zeiss Cell Observer Spinning Disk Confocal microscope equipped with 20× (NA = 0.5) objective and an environmental chamber (37 °C/5% CO_2_) at 15–30 fps over 30 to 120 s. Movies were analyzed with FIJI software to acquire fluorescence intensity (F) of 2 to 8 ROIs and 3 to 8 backgrounds (F_0_) per acquisition. Note, in Supplementary video 3, the lack of calcium transients despite beating. We observed this frequently in the no ECM control (~80% of beating areas lacked evidence of calcium transients).

## Additional Information

**How to cite this article**: Jung, J. P. *et al.* An integrated statistical model for enhanced murine cardiomyocyte differentiation via optimized engagement of 3D extracellular matrices. *Sci. Rep.* **5**, 18705; doi: 10.1038/srep18705 (2015).

## Supplementary Material

Supplementary Information

Supplementary Video S1

Supplementary Video S2

Supplementary Video S3

## Figures and Tables

**Table 1:** Experimental runs for FEs. In pattern, + and − means high and low levels, respectively. 0 indicates a center point.

Run	pattern	Col I (mg/mL)	LN (mg/mL)	FN (mg/mL)
1	−−−	0.00	0.00	0.00
2	+−−	0.83	0.00	0.00
3	−+−	0.00	0.83	0.00
4	−−+	0.00	0.00	0.83
5	−++	0.00	0.83	0.83
6	++−	0.83	0.83	0.00
7	+−+	0.83	0.00	0.83
8	+++	0.83	0.83	0.83
9	000	0.42	0.42	0.42

**Table 2:** Experimental runs for RSM, created by utilizing CCD. In pattern, A and a means the highest and lowest axial levels, respectively.

Run	pattern	Col I (mg/mL)	LN (mg/mL)	FN (mg/mL)
1	−−−	0.21	0.21	0.09
2	−−+	0.21	0.21	0.33
3	−+−	0.21	0.83	0.09
4	−++	0.21	0.83	0.33
5	+−−	0.83	0.21	0.09
6	+−+	0.83	0.21	0.33
7	++−	0.83	0.83	0.09
8	+++	0.83	0.83	0.33
9	a00	0.00	0.52	0.21
10	A00	1.04	0.52	0.21
11	0a0	0.52	0.00	0.21
12	0A0	0.52	1.04	0.21
13	00a	0.52	0.52	0.00
14	00A	0.52	0.52	0.42
15	000	0.52	0.52	0.21

**Table 3:** Experimental runs for FE with TSP1. The levels of FN were modified from 0 to 0.42 mg/mL.

Run	pattern	Col I (mg/mL)	FN (mg/mL)	TSP1 (mg/mL)
1	−−−	0.00	0.00	0.000
2	+−−	0.83	0.00	0.000
3	−+−	0.00	0.42	0.000
4	−−+	0.00	0.00	0.002
5	−++	0.00	0.42	0.002
6	++−	0.83	0.42	0.000
7	+−+	0.83	0.00	0.002
8	+++	0.83	0.42	0.002
9	000	0.42	0.21	0.001

## References

[b1] Addis R. C. & Epstein J. A. Induced regeneration-the progress and promise of direct reprogramming for heart repair. Nat Med 19, 829–836 (2013). 23836233 10.1038/nm.3225 PMC3862032

[b2] Mummery C. L. *et al.* Differentiation of human embryonic stem cells and induced pluripotent stem cells to cardiomyocytes: a methods overview. Circ Res 111, 344–358 (2012). 22821908 10.1161/CIRCRESAHA.110.227512 PMC3578601

[b3] Burridge P. W. *et al.* Chemically defined generation of human cardiomyocytes. Nat Meth 11, 855–860 (2014). 10.1038/nmeth.2999 PMC4169698 24930130

[b4] Hou P. *et al.* Pluripotent stem cells induced from mouse somatic cells by small-molecule compounds. Science 341, 651–654 (2013). 23868920 10.1126/science.1239278

[b5] Chong J. J. H. *et al.* Human embryonic-stem-cell-derived cardiomyocytes regenerate non-human primate hearts. Nature 510, 273–277 (2014). 24776797 10.1038/nature13233 PMC4154594

[b6] Baharvand H., Azarnia M., Parivar K. & Ashtiani S. K. The effect of extracellular matrix on embryonic stem cell-derived cardiomyocytes. J Mol Cell Cardiol 38, 495–503 (2005). 15733909 10.1016/j.yjmcc.2004.12.011

[b7] Parker K. K., Tan J., Chen C. S. & Tung L. Myofibrillar architecture in engineered cardiac myocytes. Circ Res 103, 340–342 (2008). 18635822 10.1161/CIRCRESAHA.108.182469 PMC3910252

[b8] Lundy S. D., Zhu W.-Z., Regnier M. & Laflamme M. A. Structural and functional maturation of cardiomyocytes derived from human pluripotent stem cells. Stem Cell Dev 22, 1991–2002 (2013). 10.1089/scd.2012.0490 PMC3699903 23461462

[b9] Flaim C. J., Chien S. & Bhatia S. N. An extracellular matrix microarray for probing cellular differentiation. Nat Meth 2, 119–125 (2005). 10.1038/nmeth736 15782209

[b10] Reticker-Flynn N. E. *et al.* A combinatorial extracellular matrix platform identifies cell-extracellular matrix interactions that correlate with metastasis. Nat Commun 3, 1122 (2012). 23047680 10.1038/ncomms2128 PMC3794716

[b11] Dolatshahi-Pirouz A. *et al.* A combinatorial cell-laden gel microarray for inducing osteogenic differentiation of human mesenchymal stem cells. Sci Rep 4 (2014). 10.1038/srep03896 PMC3905276 24473466

[b12] Ranga A. *et al.* 3D niche microarrays for systems-level analyses of cell fate. Nat Commun 5, (2014). 10.1038/ncomms5324 PMC4104440 25027775

[b13] Dawson P., Muir T., Clark-Lewis I. & Kent S. Synthesis of proteins by native chemical ligation. Science 266, 776–779 (1994). 7973629 10.1126/science.7973629

[b14] Su J., Hu B.-H., Lowe W. L. Jr, Kaufman D. B. & Messersmith P. B. Anti-inflammatory peptide-functionalized hydrogels for insulin-secreting cell encapsulation. Biomaterials 31, 308–314 (2010). 19782393 10.1016/j.biomaterials.2009.09.045 PMC2784009

[b15] Jung J. P. *et al.* ECM-incorporated hydrogels cross-linked via native chemical ligation to engineer stem cell microenvironments. Biomacromolecules 14, 3102–3111 (2013). 23875943 10.1021/bm400728e PMC3880157

[b16] Montgomery D. C. Design and Analysis of Experiments 162–349 (Wiley, 2009).

[b17] Hill T. & Lewicki P. In STATISTICS: Methods and Applications 1st edn 179–310 (StatSoft, Tusla, OK 2007).

[b18] Jung J. P., Moyano J. V. & Collier J. H. Multifactorial optimization of endothelial cell growth using modular synthetic extracellular matrices. Integr Biol 3, 185–196 (2011). 10.1039/c0ib00112k PMC3401080 21249249

[b19] Lam J., Carmichael S. T., Lowry W. E. & Segura T. Hydrogel Design of Experiments Methodology to Optimize Hydrogel for iPSC-NPC Culture. Adv Healthcare Mater 4, 534–539 (2015). 10.1002/adhm.201400410 PMC4384641 25378176

[b20] Majkut S. *et al.* Heart-specific stiffening in early embryos parallels matrix and myosin expression to optimize beating. Curr Biol 23, 2434–2439 (2013). 24268417 10.1016/j.cub.2013.10.057 PMC4116639

[b21] Young J. L. & Engler A. J. Hydrogels with time-dependent material properties enhance cardiomyocyte differentiation *in vitro*. Biomaterials 32, 1002–1009 (2011). 21071078 10.1016/j.biomaterials.2010.10.020 PMC3000555

[b22] Hanson K. P. *et al.* Spatial and temporal analysis of extracellular matrix proteins in the developing murine heart: a blueprint for regeneration. Tissue Eng Part A 19, 1132–1143 (2012). 23273220 10.1089/ten.tea.2012.0316 PMC3609645

[b23] Sa S., Wong L. & McCloskey K. E. Combinatorial fibronectin and laminin signaling promote highly efficient cardiac differentiation of human embryonic stem cells. BioRes Open Access 3, 150–161 (2014). 25126479 10.1089/biores.2014.0018 PMC4120929

[b24] Laperle A., Masters K. S. & Palecek S. P. Influence of substrate composition on human embryonic stem cell differentiation and extracellular matrix production in embryoid bodies. Biotechnol Prog 31, 212–219 (2015). 25311359 10.1002/btpr.2001 PMC4340738

[b25] Gunning P. *et al.* Differential patterns of transcript accumulation during human myogenesis. Mol Cell Biol 7, 4100–4114 (1987). 3431550 10.1128/mcb.7.11.4100-4114.1987 PMC368081

[b26] Wade R. *et al.* Regulation of contractile protein gene family mRNA pool sizes during myogenesis. Dev Biol 142, 270–282 (1990). 2257967 10.1016/0012-1606(90)90348-m

[b27] Sutherland C. J., Elsom V. L., Gordon M. L., Dunwoodie S. L. & Hardeman E. C. Coordination of skeletal muscle gene expression occurs late in mammalian development. Dev Biol 146, 167–178 (1991). 2060700 10.1016/0012-1606(91)90457-e

[b28] Martin A. F. Turnover of cardiac troponin subunits. Kinetic evidence for a precursor pool of troponin-I. J Biol Chem 256, 964–968 (1981). 7451483

[b29] Nii M. *et al.* The effects of interactive mechanical and biochemical niche signaling on osteogenic differentiation of adipose-derived stem cells using combinatorial hydrogels. Acta Biomater 9, 5475–5483 (2013). 23153761 10.1016/j.actbio.2012.11.002

[b30] Kijlstra J. D. *et al.* Integrated analysis of the contractile kinetics, force generation, and electrical activity in single human pluripotent stem cell derived cardiomyocytes. *Stem Cell Report* (in press) 10.1016/j.stemcr.2015.10.017 PMC4682285 26626178

[b31] Zhu W.-Z., Santana L. F. & Laflamme M. A. Local control of excitation-contraction coupling in human embryonic stem cell-derived cardiomyocytes. PLoS ONE 4, e5407 (2009). 19404384 10.1371/journal.pone.0005407 PMC2671137

[b32] Itzhaki I. *et al.* Calcium handling in human induced pluripotent stem cell derived cardiomyocytes. PLoS ONE 6, e18037 (2011). 21483779 10.1371/journal.pone.0018037 PMC3069979

[b33] Krishna S. M. & Golledge J. The role of thrombospondin-1 in cardiovascular health and pathology. Int J Cardiol 168, 692–706 (2013). 23664438 10.1016/j.ijcard.2013.04.139

[b34] Chung C., Pruitt B. L. & Heilshorn S. C. Spontaneous cardiomyocyte differentiation of mouse embryoid bodies regulated by hydrogel crosslink density. Biomater Sci 1, 1082–1090 (2013). 24748962 10.1039/C3BM60139K PMC3987919

[b35] Schukur L., Zorlutuna P., Cha J. M., Bae H. & Khademhosseini A. Directed differentiation of size-controlled embryoid bodies towards endothelial and cardiac lineages in RGD-modified poly (ethylene glycol) hydrogels. Adv Healthcare Mater 2, 195–205 (2013). 10.1002/adhm.201200194 PMC3635117 23193099

[b36] Lian X. *et al.* Robust cardiomyocyte differentiation from human pluripotent stem cells via temporal modulation of canonical Wnt signaling. Proc Natl Acad Sci USA 109, E1848–E1857 (2012). 22645348 10.1073/pnas.1200250109 PMC3390875

[b37] Battista S. *et al.* The effect of matrix composition of 3D constructs on embryonic stem cell differentiation. Biomaterials 26, 6194–6207 (2005). 15921736 10.1016/j.biomaterials.2005.04.003

[b38] Singelyn J. M. *et al.* Naturally derived myocardial matrix as an injectable scaffold for cardiac tissue engineering. Biomaterials 30, 5409–5416 (2009). 19608268 10.1016/j.biomaterials.2009.06.045 PMC2728782

[b39] Lockhart M., Wirrig E., Phelps A. & Wessels A. Extracellular matrix and heart development. Birth Def Res A Clin Mol Teratol 91, 535–550 (2011). 10.1002/bdra.20810 PMC3144859 21618406

[b40] Ng A. *et al.* Loss of glypican-3 function causes growth factor-dependent defects in cardiac and coronary vascular development. Dev Biol 335, 208–215 (2009). 19733558 10.1016/j.ydbio.2009.08.029 PMC2763964

[b41] Wirrig E. E. *et al.* Cartilage link protein 1 (Crtl1), an extracellular matrix component playing an important role in heart development. Dev Biol 310, 291–303 (2007). 17822691 10.1016/j.ydbio.2007.07.041 PMC2254939

[b42] Norris R. A. *et al.* Identification and detection of the periostin gene in cardiac development. Anat Rec A Discov Mol Cell Evol Biol 281A, 1227–1233 (2004). 15532025 10.1002/ar.a.20135

[b43] Hodgkinson C. *et al.* Abi3bp regulates cardiac progenitor cell proliferation and differentiation. Circ Res 115, 1007–1016 (2014). 25296984 10.1161/CIRCRESAHA.115.304216 PMC4258122

[b44] Barry W. H. & Bridge J. H. Intracellular calcium homeostasis in cardiac myocytes. Circulation 87, 1806–1815 (1993). 8389258 10.1161/01.cir.87.6.1806

[b45] Xi J. *et al.* Comparison of contractile behavior of native murine ventricular tissue and cardiomyocytes derived from embryonic or induced pluripotent stem cells. FASEB J 24, 2739–2751 (2010). 20371616 10.1096/fj.09-145177

[b46] Lawler J., Weinstein R. & Hynes R. O. Cell attachment to thrombospondin: the role of ARG-GLY-ASP, calcium, and integrin receptors. J Cell Biol 107, 2351–2361 (1988). 2848850 10.1083/jcb.107.6.2351 PMC2115659

