# Supplementary material for: An integrated statistical model for enhanced murine cardiomyocyte differentiation via optimized engagement of 3D extracellular matrices
Source: Sci Rep. 2015 Dec 21;5:18705. doi: 10.1038/srep18705 (PMC4685314; doi:10.1038/srep18705)
Supplement: Supplementary Information [file srep18705-s1.doc]

**Supplementary Information**

An integrated statistical model for enhanced murine cardiomyocyte differentiation via optimized engagement of 3D extracellular matrices

Jangwook P. Jung^1,2^, Dongjian Hu^3^, Ibrahim J. Domian^3^ and Brenda M. Ogle^1,2,4,5,6*^

Author Affiliation: 1) Department of Biomedical Engineering, 2) Stem Cell Institute, 3) Cardiovascular Research Center, Massachusetts General Hospital & Harvard Medical School, Boston, MA 02114 U.S.A., 4) Masonic Cancer Center, 5) Lillehei Heart Institute, 6) Institute for Engineering in Medicine, University of Minnesota – Twin Cities, Minneapolis, MN 55455, U.S.A.

**Supplementary Figures**

*Quantification of Stiffness of Composites*

An ARES-LS2 rheometer (TA Instruments, New Castle, DE) was used to measure the viscoelasticity of PEG hydrogels or acellular ECM composites. The upper plate (parallel plate, 8 mm diameter) was lowered until it was in conformal contact with the top surface of PEG hydrogels or acellular ECM composites. Frequency sweeping measurements were performed from 0.1 to 10 Hz at 1.0% strain, and three independent samples were tested at each condition.


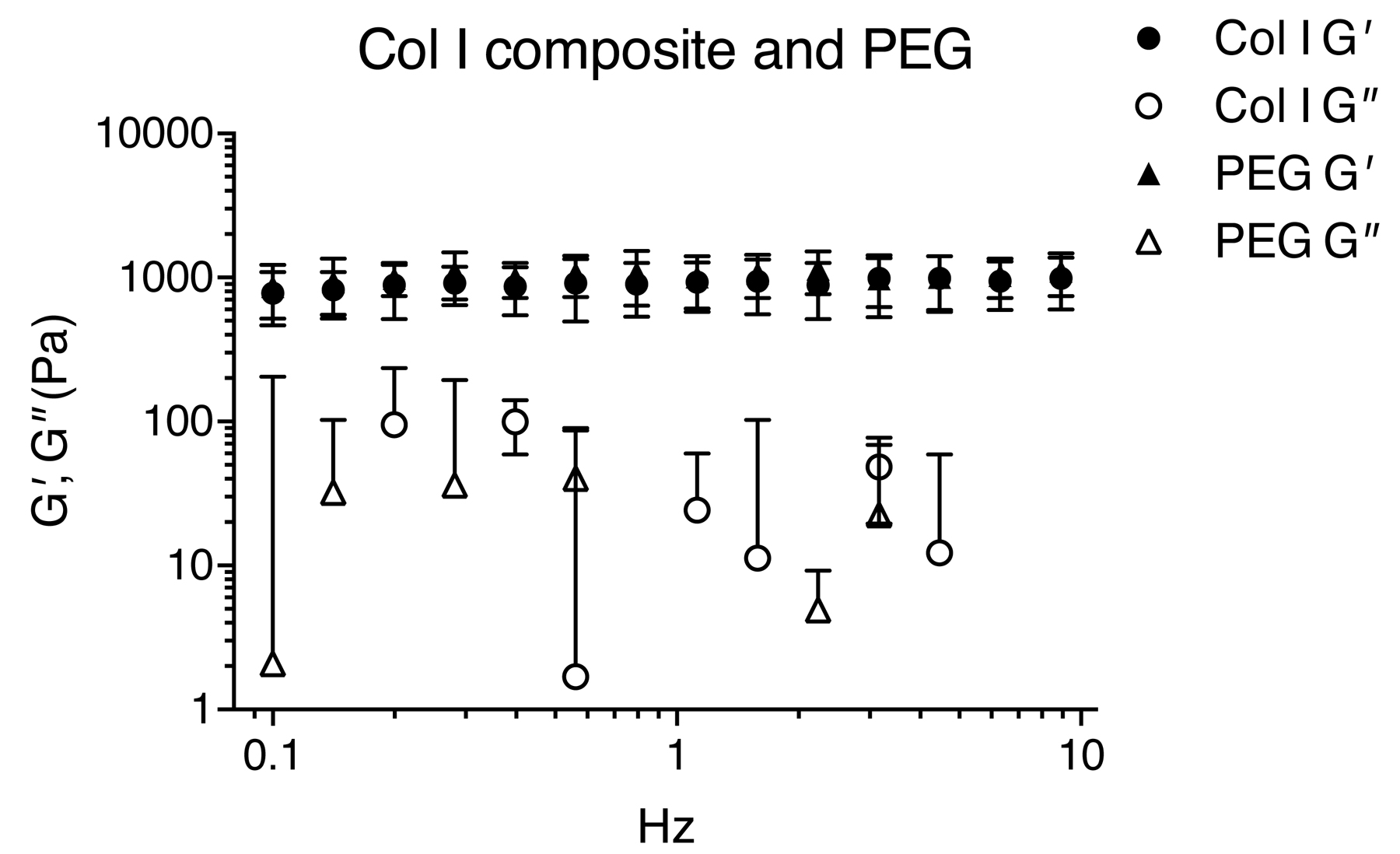


**Figure S1.** Oscillating rheometry of Col I composite (Col I concentration = 2.5 mg/mL) and PEG (no ECM control). The extensive overlap indicates that storage moduli (G′) of Col I was not significantly different from those of PEG alone. G″ (loss modulus), mean ± S.D., n=3. The final concentration of PEG was 40 mg/mL.


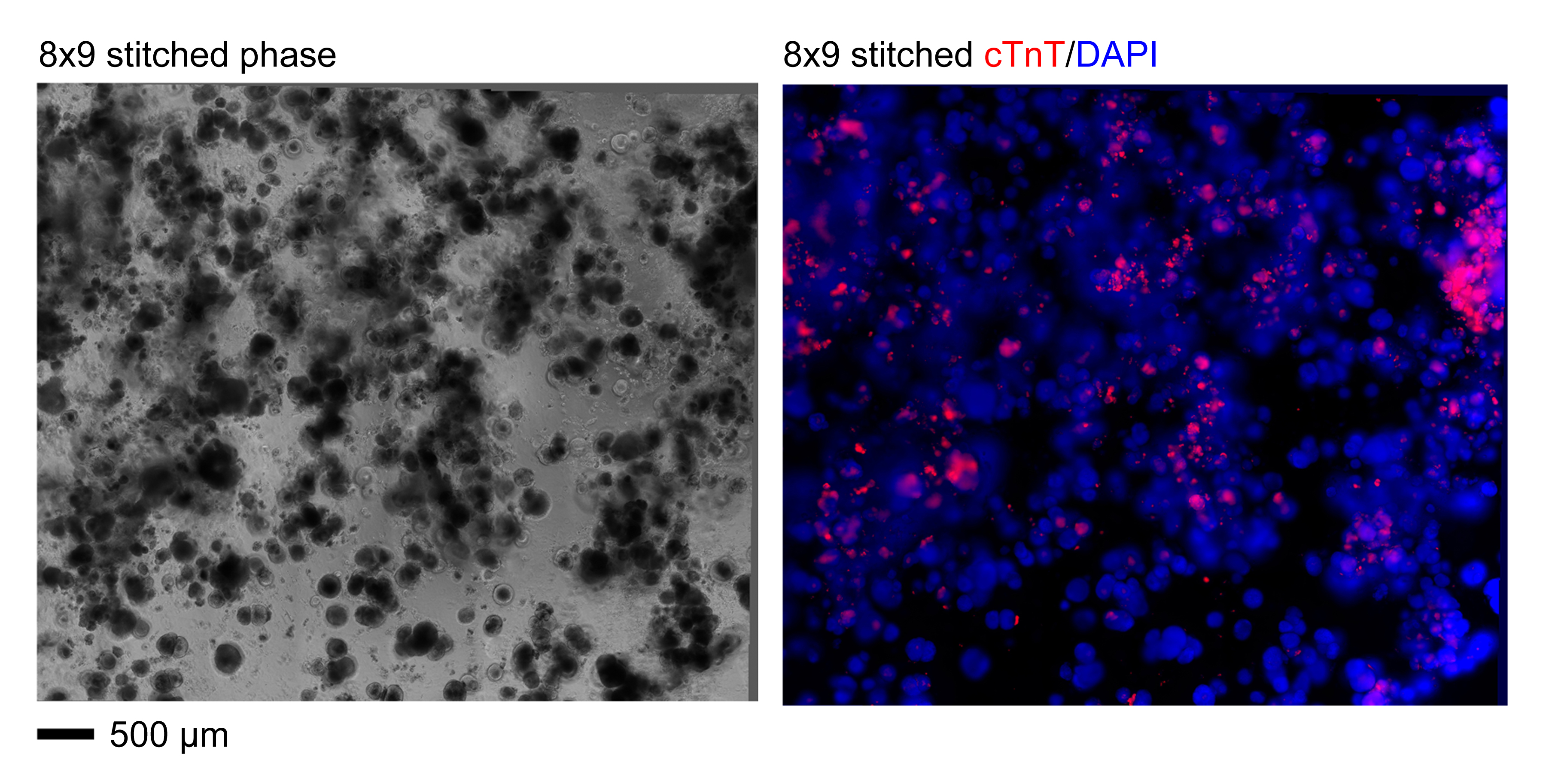
**Figure S2.** Eight by nine stitched fields of view of phase contrast microscopy (left) and immunofluorescence staining (right) of differentiated miPSCs in one entire ECM composite (formulation 000 in FE) after 21 days of culture. Red: cTnT, blue: DAPI. Scale bar: 500 µm


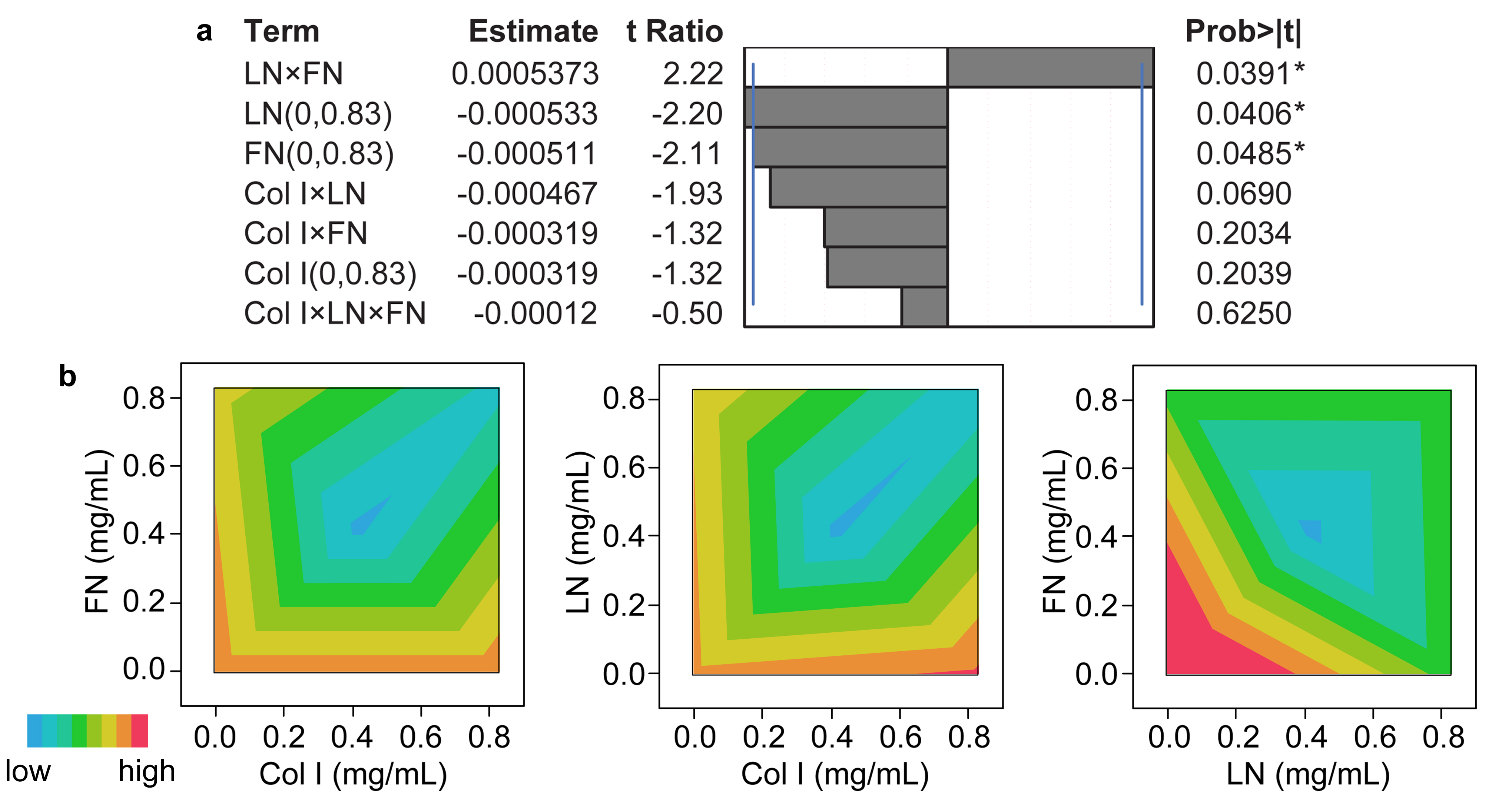


**Figure S3. (a)** Effect magnitudes of all main factors, two-factor interactions, and the three-factor interaction for the expression of Tnnt2 (gene). Results were calculated by FE. Blue lines indicate significant probability values of 0.05. **(b)** Contour plots showing the interactions between FN and Col I, LN and Col I, and FN and LN.

**
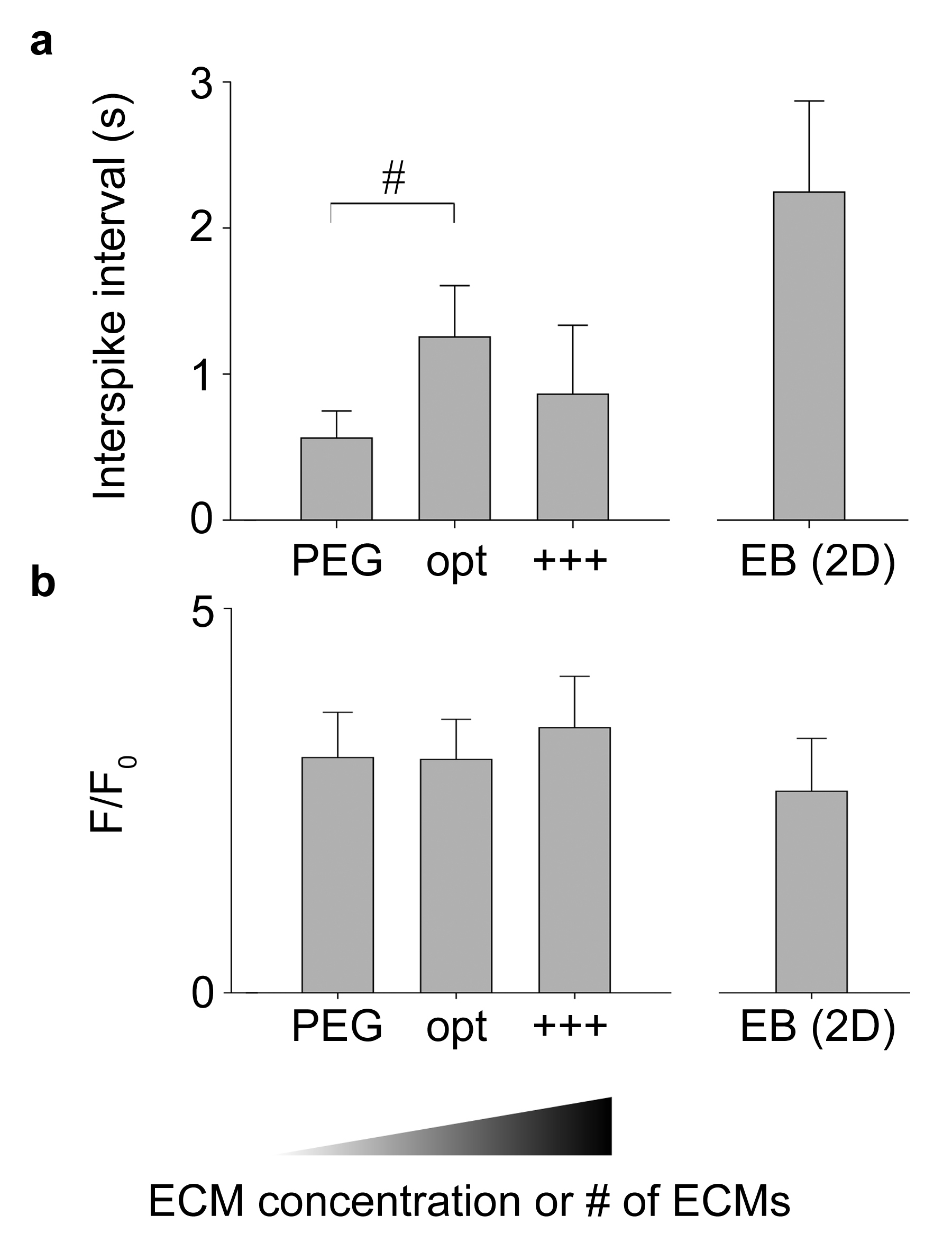
**

**Figure S4.** (a) Interspike intervals (ISI) of Ca^2+^ transients were calculated in ECM composites and EB (2D) control. ANOVA Tukey’s HSD *post hoc* test, n=5 ^#^p<0.05, mean ± S.D. (b) Fluo-4 AM (Ca^2+^ indicator) intensity (F) was normalized to the baseline fluorescence (F_0_).

**
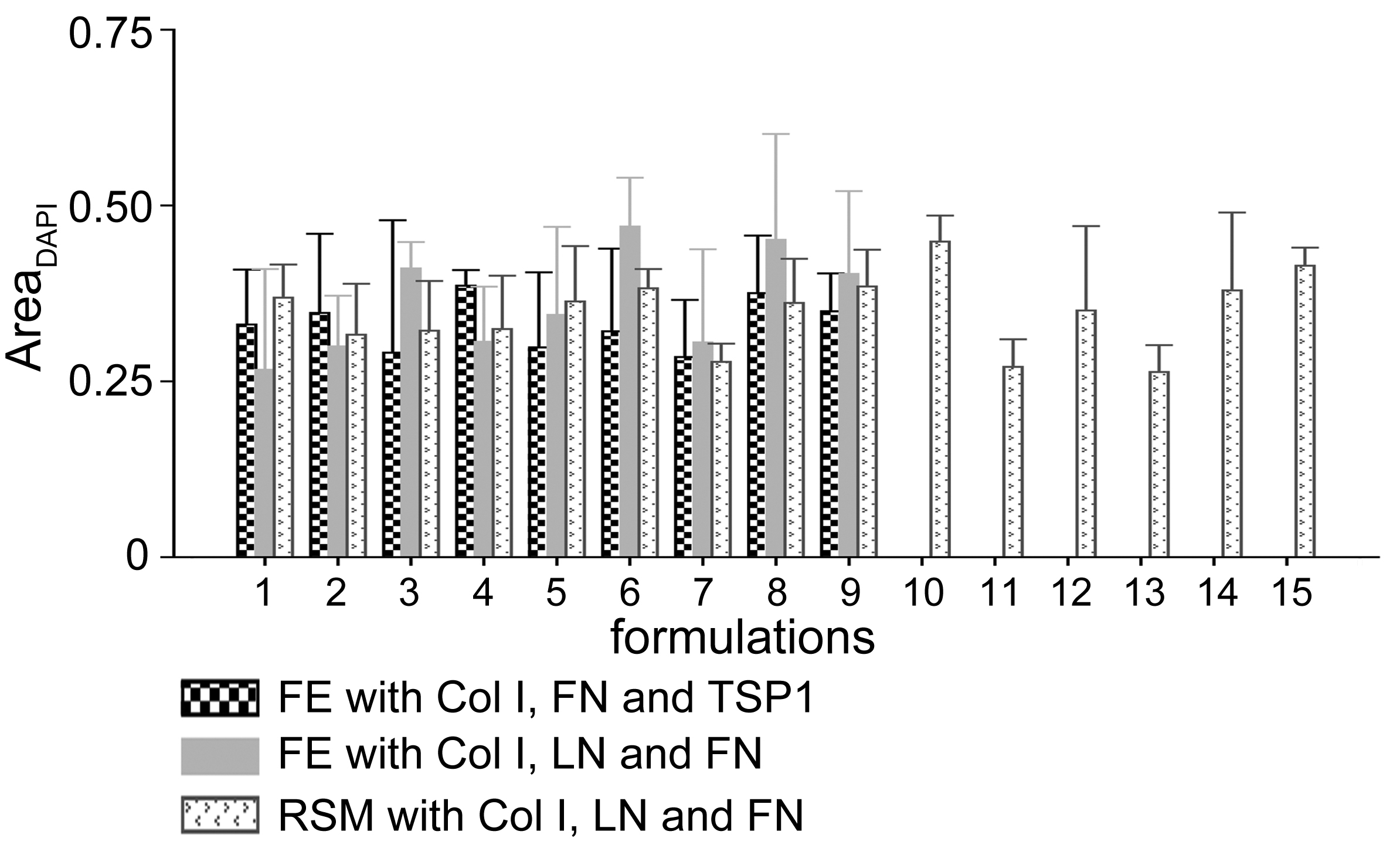
**

**Figure S5.** The number of miPSCs encapsulated in TSP1 containing ECM composites were maintained, similar to ECM composites containing Col I, LN, and FN, after 21 days in culture. Graph shows a fraction of area containing DAPI staining for ECM composites of FEs and RSM. Formulation details shown in Tables of 1, 2, and 3.

**Supplementary Tables**

**Table S1.** The results of response surface regression that yielded the coefficients of the response surface.

|  | |  |  |  |
| --- | --- | --- | --- | --- |
|  | Col I | LN | FN | cTnT (area) |
| Col I | -0.009876 | -0.060129 | -0.059199 | 0.0344855 |
| LN |  | -0.013269 | -0.018542 | 0.0750568 |
| FN |  |  | 0.0493484 | 0.0378275 |

**Table S2.** The eigenvalues and eigenvectors (canonical curvature) of the response surface were calculated from the response surface regression.

|  |  |  |  |
| --- | --- | --- | --- |
| Eigenvalue | 0.0617 | 0.0138 | -0.0494 |
| Col I | -0.3981 | 0.58844 | 0.70374 |
| LN | 0.04633 | -0.75328 | 0.65607 |
| FN | 0.91617 | 0.29379 | 0.27262 |

**Table S3.** The response surface regression calculated a set of three ECM protein formulation.

| Variable | Critical Value (mg/mL) |
| --- | --- |
| Col I | 1.1495487 |
| LN | 0.4490198 |
| FN | 0.2766226 |
|  |  |

**Table S4.** ECM composites with these formulations were produced to test the enhancement of sarcomeric protein expression, contractile kinetics and Ca^2+^ handling.

| Run | pattern | Col I (mg/mL) | LN (mg/mL) | FN (mg/mL) |
| --- | --- | --- | --- | --- |
| 1 | PEG | 0.00 | 0.00 | 0.00 |
| 2 | opt | 1.15 | 0.45 | 0.28 |
| 3 | +++ | 0.83 | 0.83 | 0.33 |

**Legends for the Video files**

Supplementary video 1. Ca^2+^ transients in an ECM composite of the optimal formulation over 30 s. The frame rate was approximately 18.3 fps.

Supplementary video 2. Ca^2+^ transients in an ECM composite of the +++ formulation (see Table 3 in the main text) over 30 s. The frame rate was approximately 18.3 fps.

Supplementary video 3. A PEG hydrogel (no ECM control) showed no Ca^2+^ transients with beating. The frame rate was approximately 18.3 fps. Lack of Ca^2+^ transients in beating areas was common in the no ECM control (~80% of beating areas).
